# Supplementary material for: Mid-gestational cell-type-specific transcriptomic signatures in the prefrontal and superior temporal cortex in Down syndrome
Source: Nat Commun. 2025 Dec 11;16:11249. doi: 10.1038/s41467-025-66109-9 (PMC12717210; doi:10.1038/s41467-025-66109-9)
Supplement: Supplementary file 1 — Supplementary Information [file 41467_2025_66109_MOESM1_ESM.pdf]

Supplementary Information for

Mid-gestational cell-type specific transcriptomic signatures in the prefrontal and superior temporal cortex in Down syndrome

Niu et al.

This PDF file includes:

Supplementary Fig. 1 to 15

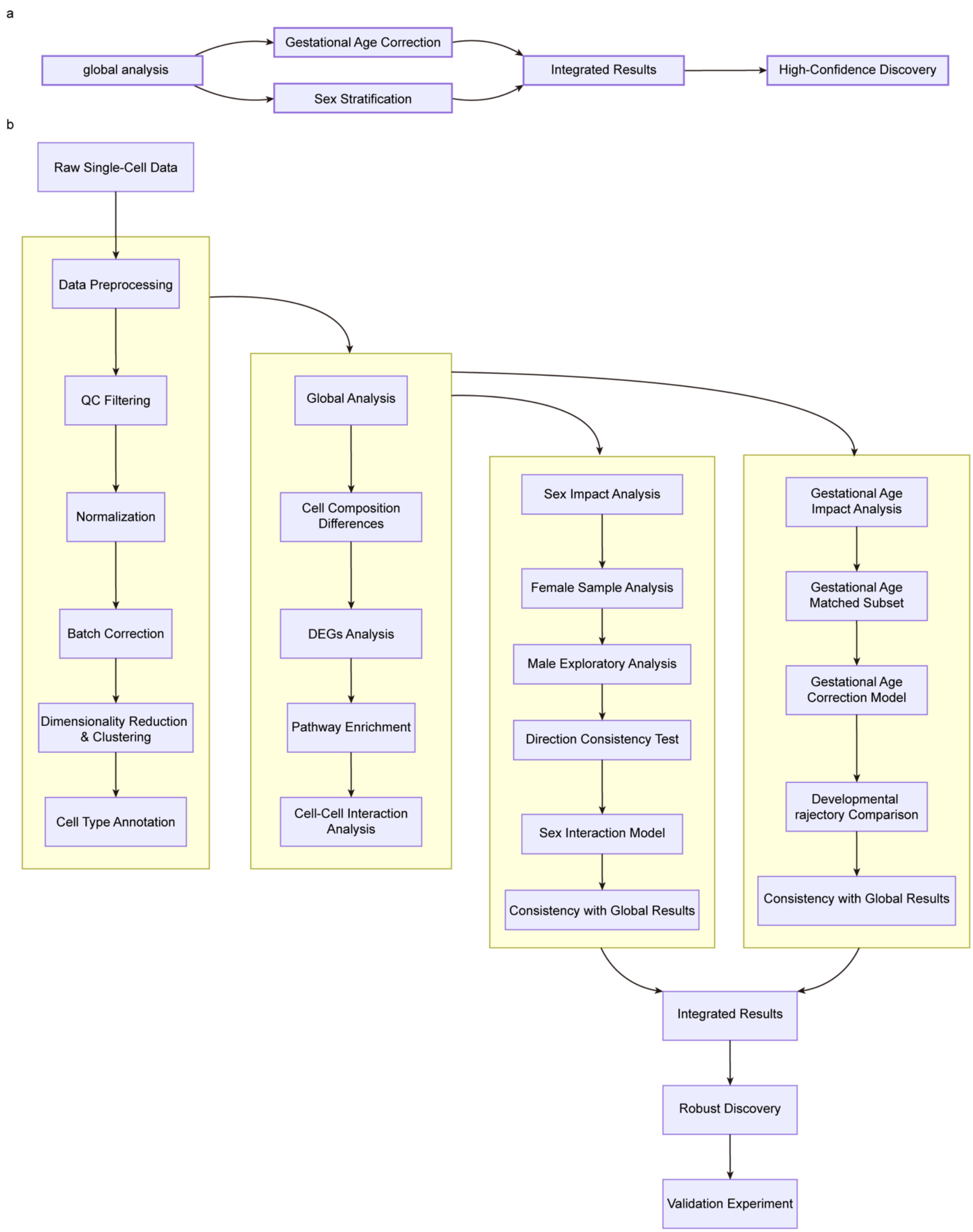

Supplementary Fig. 1. Data analysis framework. a Data analysis process based on gestational age and gender stratification. **b** Detailed process of data analysis.

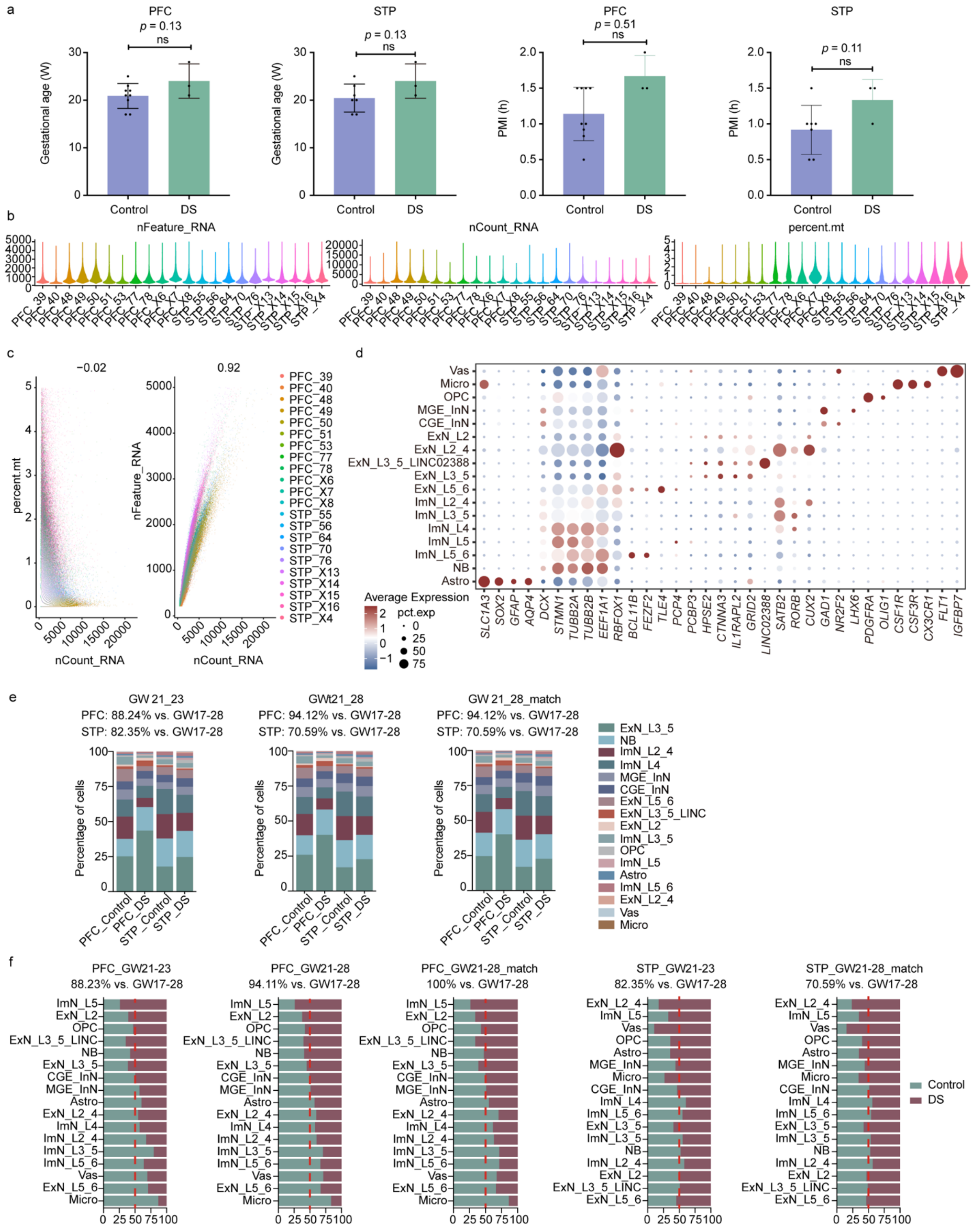

Supplementary Fig. 2. Quality control and cell proportion changes in the cortex of patients with DS. a The intergroup differences in gestational age and PMI of the samples (two-sided independent-sample t-test).

n = 9 biological replicates for PFC_Control, *n* = 3 biological replicates for PFC_DS, *n* = 7 biological replicates for STP_Control, *n* = 3 biological replicates for STP_DS. **b** Violin plots show 15 snRNA-seq data quality of nFeature_RNA, nCount_RNA, percent.mt. **c** Left: Scatter plot showing the relationship between the total UMI counts (nCount_RNA) and the proportion of mitochondrial genes (percent.mt) for each cell across different samples. Each dot represents a single cell, colored by sample. A low proportion of mitochondrial genes (< 5%) indicates good quality cells, while higher values may suggest stressed or dying cells. Right: Scatter plot showing the correlation between the total UMI counts (nCount_RNA) and the number of detected genes (nFeature_RNA) for each cell across different samples. Each dot represents a single cell, colored by sample. The high correlation (R = 0.92) suggests consistent detection of features across cells, which indicates data quality. **d** Bubble dot plots of the cell specific marker genes. The size of the dot indicates expression percentage and the darkness of the color indicates average expression. **e** The cellular composition of PFC and STP illustrates a comparative analysis of cell proportions between DS and control groups from different stratified data. **f** Bar plot showing the comparison of various cell types in different stratified data of PFC and STP.

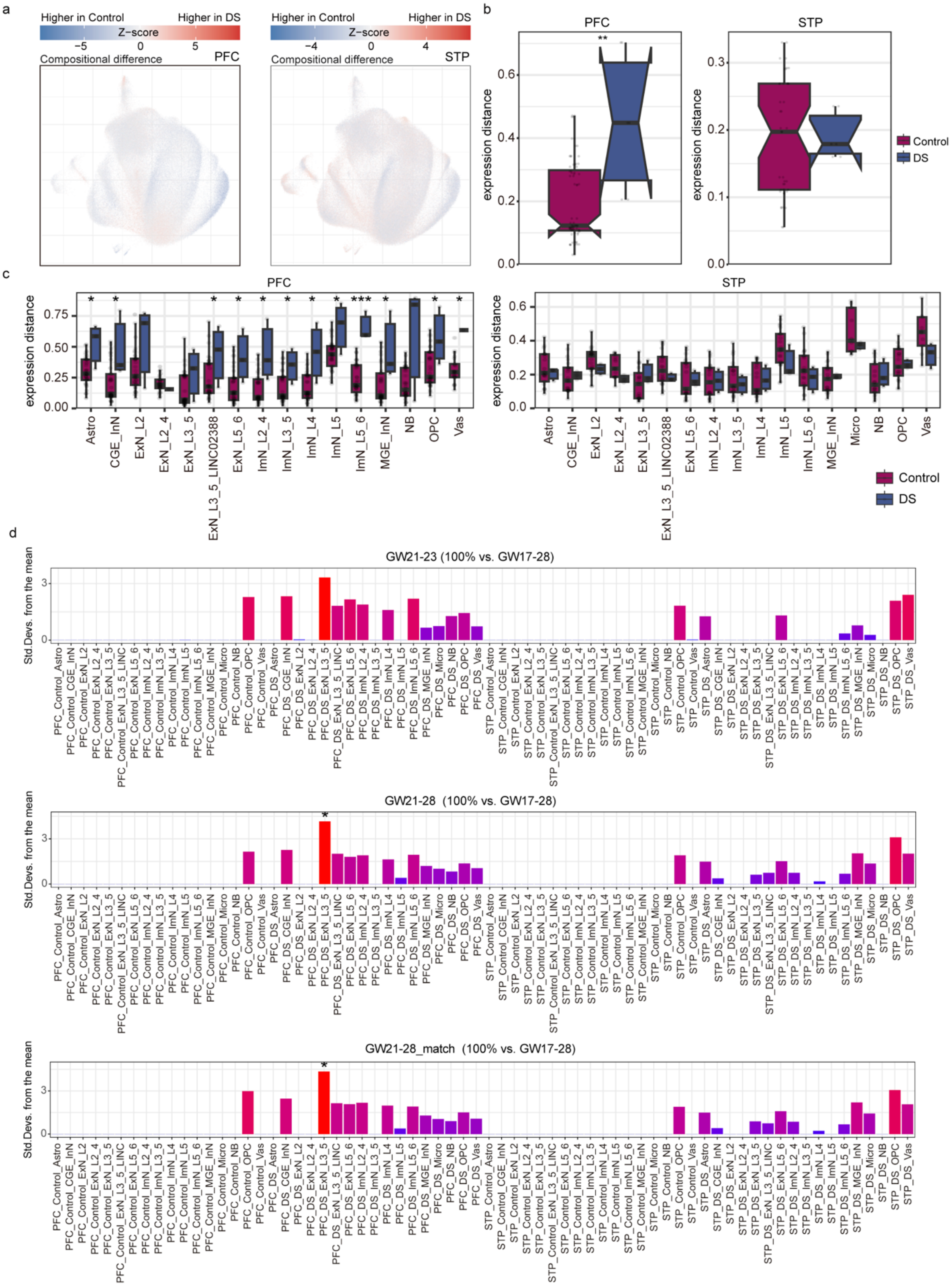

Supplementary Fig. 3. Transcriptomic changes in the cortex of patients with DS. a Statistical assessment of the cell density differences in PFC and STP. Student's t test was used, visualized as a z score. Red indicates

upregulation in DS, and blue indicates downregulation. **b** Boxplots showing interindividual gene expression distances (based on Pearson correlation) within control and DS samples from PFC and STP, averaged across all neuronal cell types. $^{**}p = 0.001$ to 0.01. **c** Boxplots showing interindividual gene expression distances for different cell types within control and DS samples from PFC and STP. $^{*}p = 0.01$ to 0.05. **d** Enrichment levels of chromosome 21 genes in PFC and STP for both control and DS groups from different stratified data (two-sided bootstrap-based test). $^{*}p = 0.01$ to 0.05. Percentages indicate the consistency rate of each stratified subgroup compared to the full dataset (GW17–28).

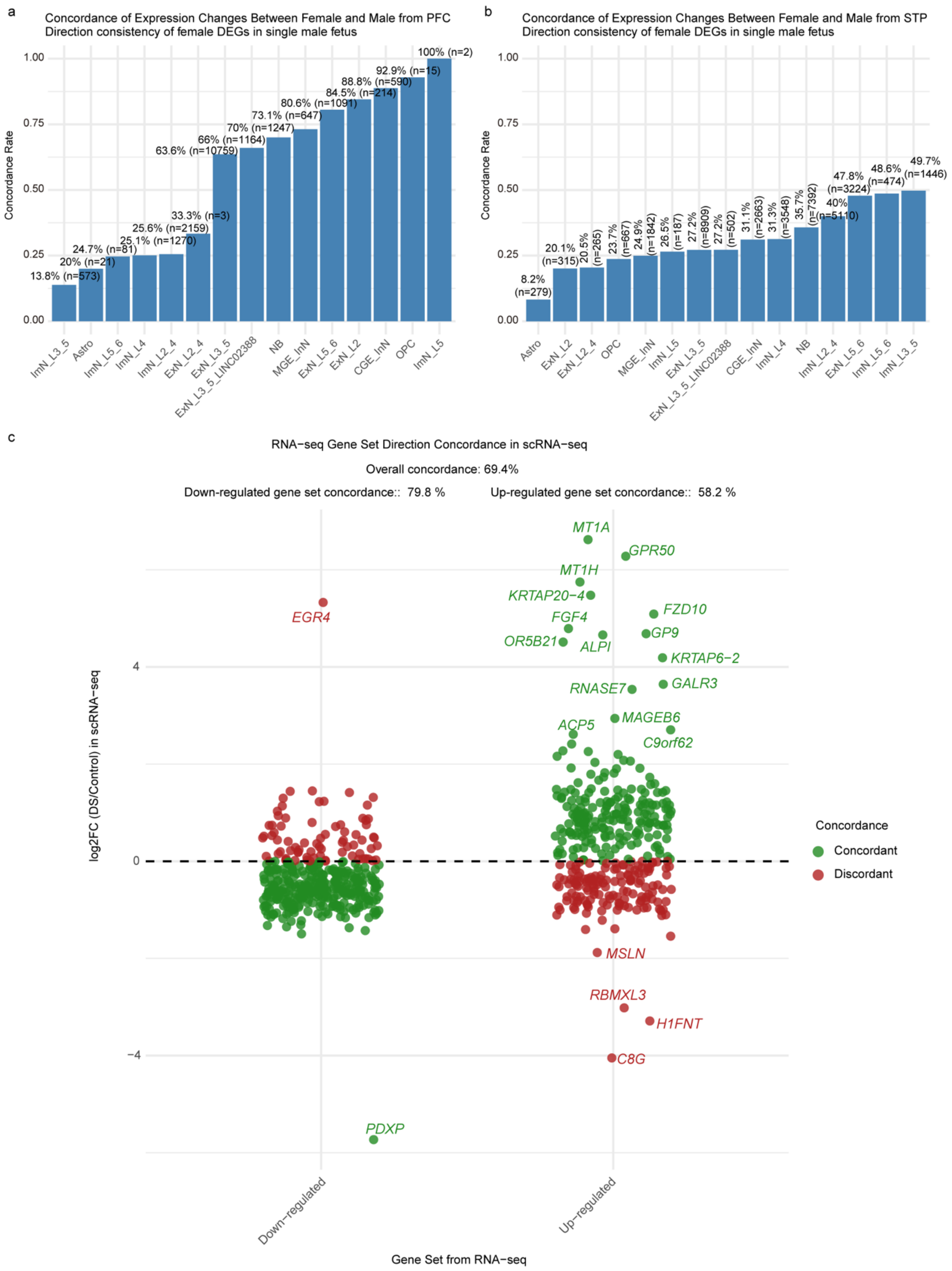

Supplementary Fig. 4. Consistency comparison of DEGs between male and female samples. a-b Concordance of expression changes between female and male from PFC (**a**) and STP (**b**). **c** The direction

consistency of DEGs of Bulk RNA-seq in DS snRNA-seq data. Percentages indicate the consistency rate of each stratified subgroup compared to the full dataset (GW17–28).

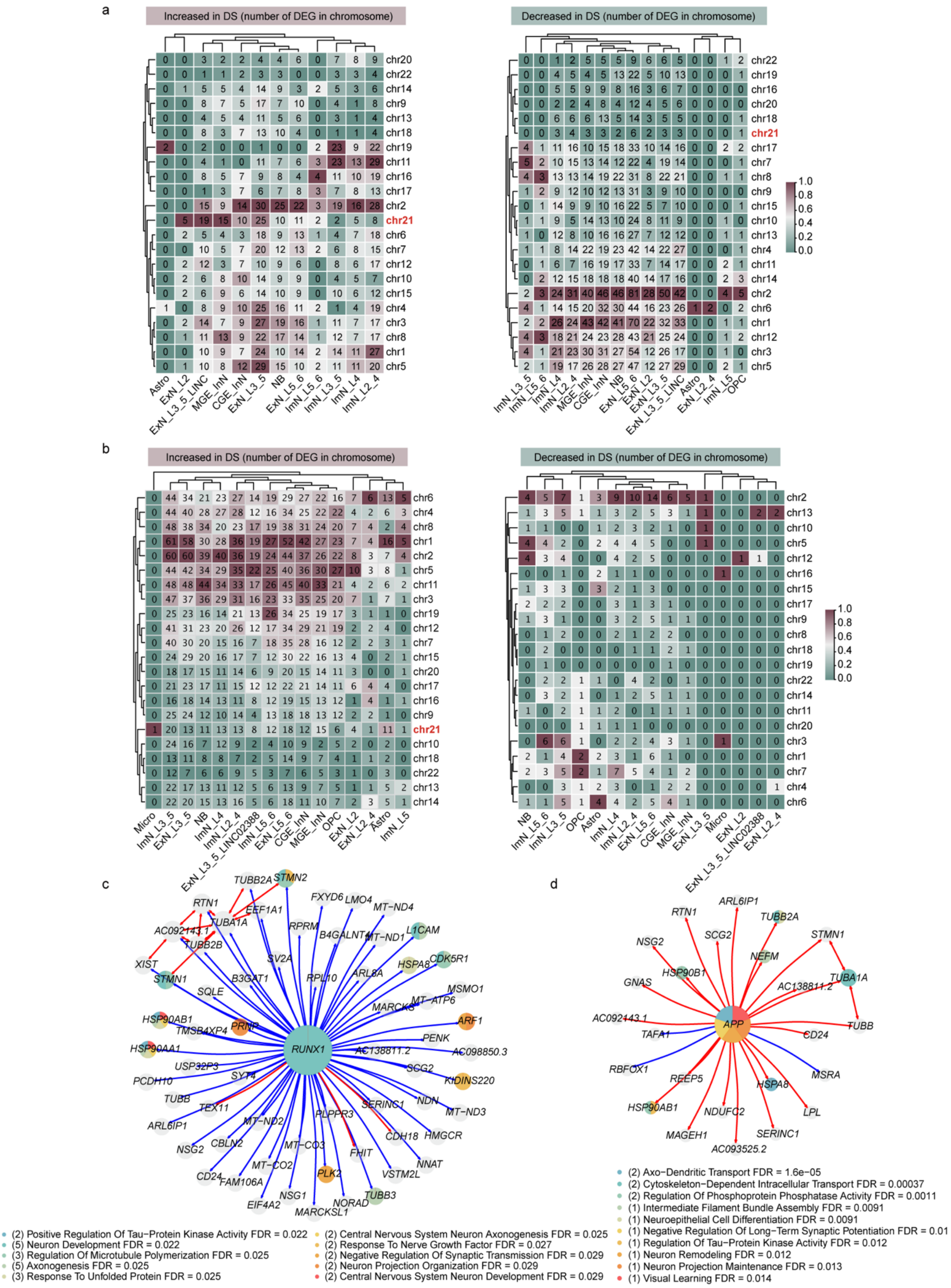
a
Increased in DS (number of DEG in chromosome)
Decreased in DS (number of DEG in chromosome)
chr20
chr22
chr14
chr9
chr13
chr18
chr19
chr11
chr16
chr17
chr2
chr21
chr6
chr7
chr12
chr10
chr15
chr4
chr3
chr8
chr1
chr5
Astro
ExN_L2
ExN_L3_5_LINC
MGE_InN
CGE_InN
ExN_L3_5
NB
ExN_L5_6
ImN_L5_6
ImN_L3_5
ImN_L4
ImN_L2_4
Micro
OPC
1.0
0.8
0.6
0.4
0.2
0.0
b
Increased in DS (number of DEG in chromosome)
Decreased in DS (number of DEG in chromosome)
ExN_L3_5_LINC02388
ExN_L2_4
c
RUNX1
d
APP
(2) Positive Regulation Of Tau−Protein Kinase Activity FDR = 0.022
(5) Neuron Development FDR = 0.022
(3) Regulation Of Microtubule Polymerization FDR = 0.025
(5) Axonogenesis FDR = 0.025
(3) Response To Unfolded Protein FDR = 0.025
(2) Central Nervous System Neuron Axonogenesis FDR = 0.025
(2) Response To Nerve Growth Factor FDR = 0.027
(2) Negative Regulation Of Synaptic Transmission FDR = 0.029
(2) Neuron Projection Organization FDR = 0.029
(2) Central Nervous System Neuron Development FDR = 0.029
(2) Axo−Dendritic Transport FDR = 1.6e−05
(2) Cytoskeleton−Dependent Intracellular Transport FDR = 0.00037
(2) Regulation Of Phosphoprotein Phosphatase Activity FDR = 0.0011
(1) Intermediate Filament Bundle Assembly FDR = 0.0091
(1) Neuroepithelial Cell Differentiation FDR = 0.0091
(1) Negative Regulation Of Long−Term Synaptic Potentiation FDR = 0.01
(1) Regulation Of Tau−Protein Kinase Activity FDR = 0.012
(1) Neuron Remodeling FDR = 0.012
(1) Neuron Projection Maintenance FDR = 0.013
(1) Visual Learning FDR = 0.014

Supplementary Fig. 5. The distribution of DEGs on chromosomes from the DS cortex. a, b The heat map shows the distribution of DEGs of different cell types in the PFC (a) and STP (b) on chromosomes. The numbers represent the absolute number of DEGs on the chromosomes. **c, d** The egocentric plot shows the connections between the KO gene (*RUNX1* and *APP*) and significant virtual KO perturbed genes (FDR < 0.05). Nodes are color-coded by each gene's member- ship association with enriched functional groups, as reported in the Enrichr analysis. The displaying gene sets are selected - only those with functions related to the Mendelian disease phenotype are shown.

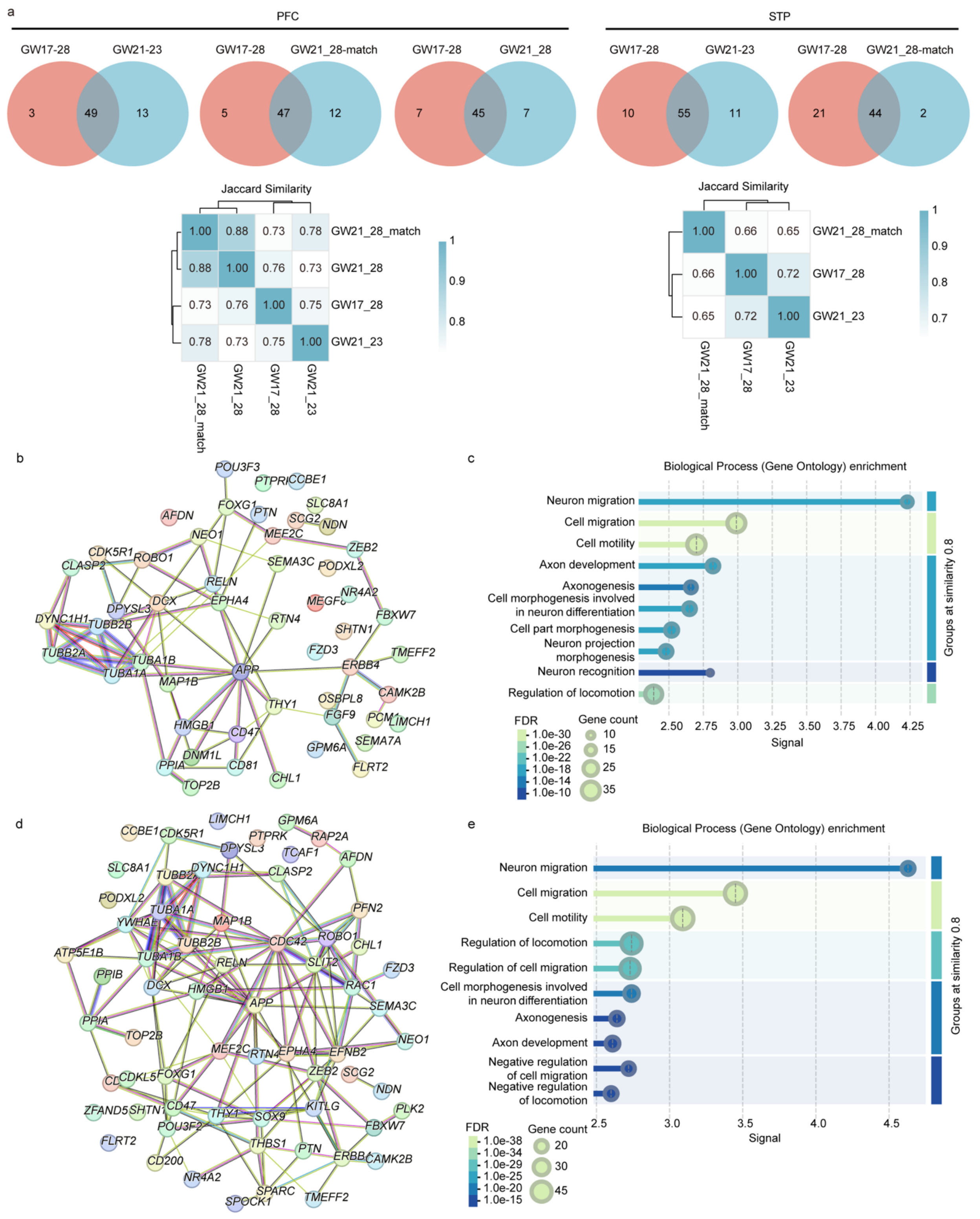

Supplementary Fig. 6. Differential expression of neuronal migration genes in the PFC and STP of patients with DS. a The Venn diagram shows abnormal expression of multiple neuron migration genes. Pink represents

the differentially expressed cell migration genes in the global analysis. Blue represents the differentially expressed cell migration genes in different stratified data. Jaccard similarity analyses compared overlapped migration-related genes between stratified subgroups to the full GW17–28 dataset. **b** The PPI network shows the close connections among the 52 differentially expressed migration genes from PFC. **c** Functional enrichment analysis of the 52 differentially expressed migration genes from PFC. **d** The PPI network shows the close connections among the 65 differentially expressed migration genes from STP. **e** Functional enrichment analysis of the 65 differentially expressed migration genes from STP.

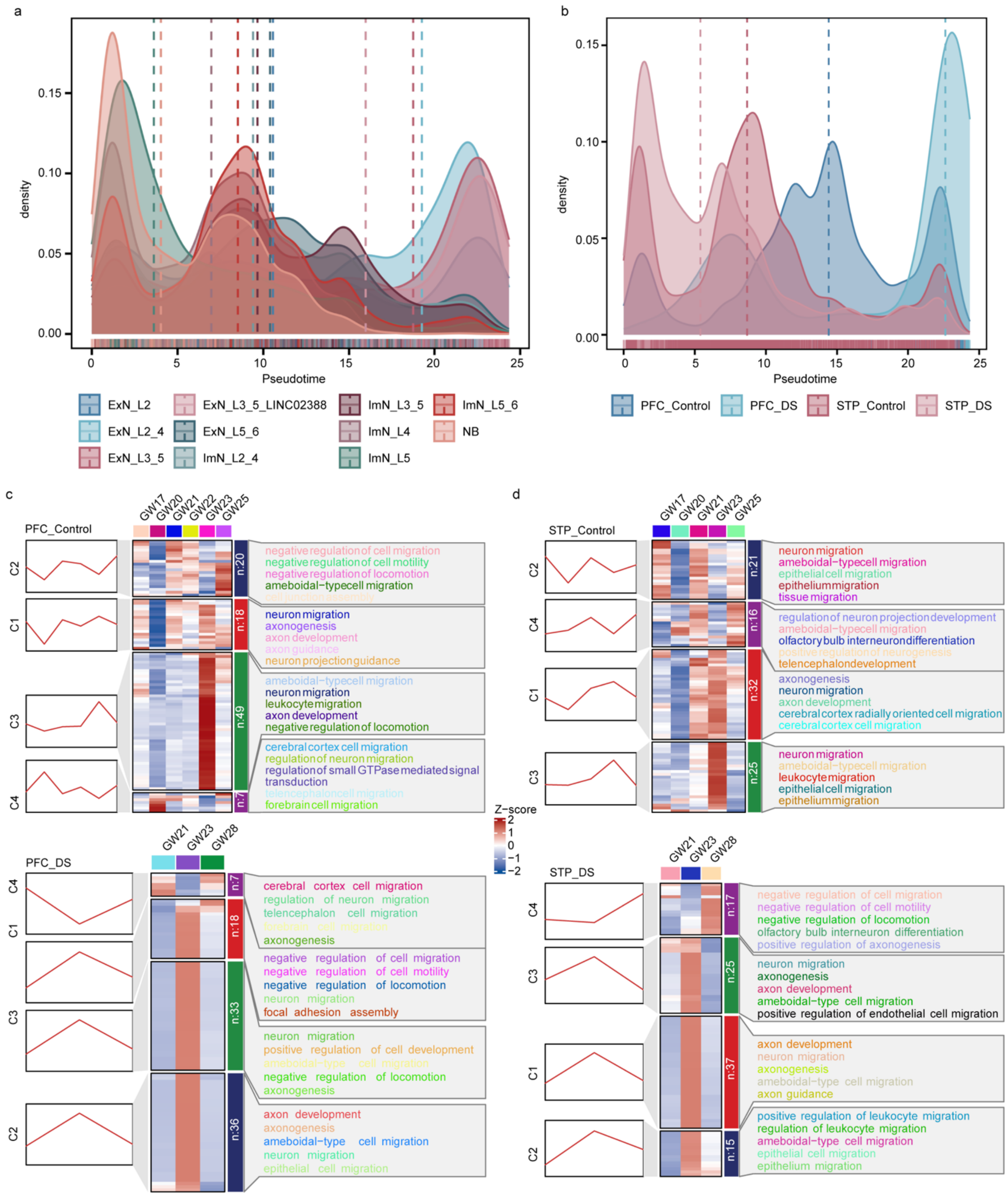

Supplementary Fig. 7. Pseudotime and timing-series analysis of neuron migration gene clusters in neuron across gestational age. a, b Density plot showing the distribution of ExN subclusters (a) and group (b) along the trajectory. **c** This figure presents gestational age-associated transcriptional changes of neuron migration gene in PFC ExN, with heatmaps and gene ontology (GO) enrichment terms. Gene clusters (C1, C2, C3, C4) represent

distinct expression patterns and functional enrichment. Heatmaps display Z-scores of gene expression for age-dependent clusters across. Red indicates upregulation, blue indicates downregulation. GO Enrichment Terms: Biological processes (BPs) enriched within each gene cluster are displayed next to the heatmaps. **d** This figure presents gestational age-associated transcriptional changes of neuron migration gene in STP ExN.

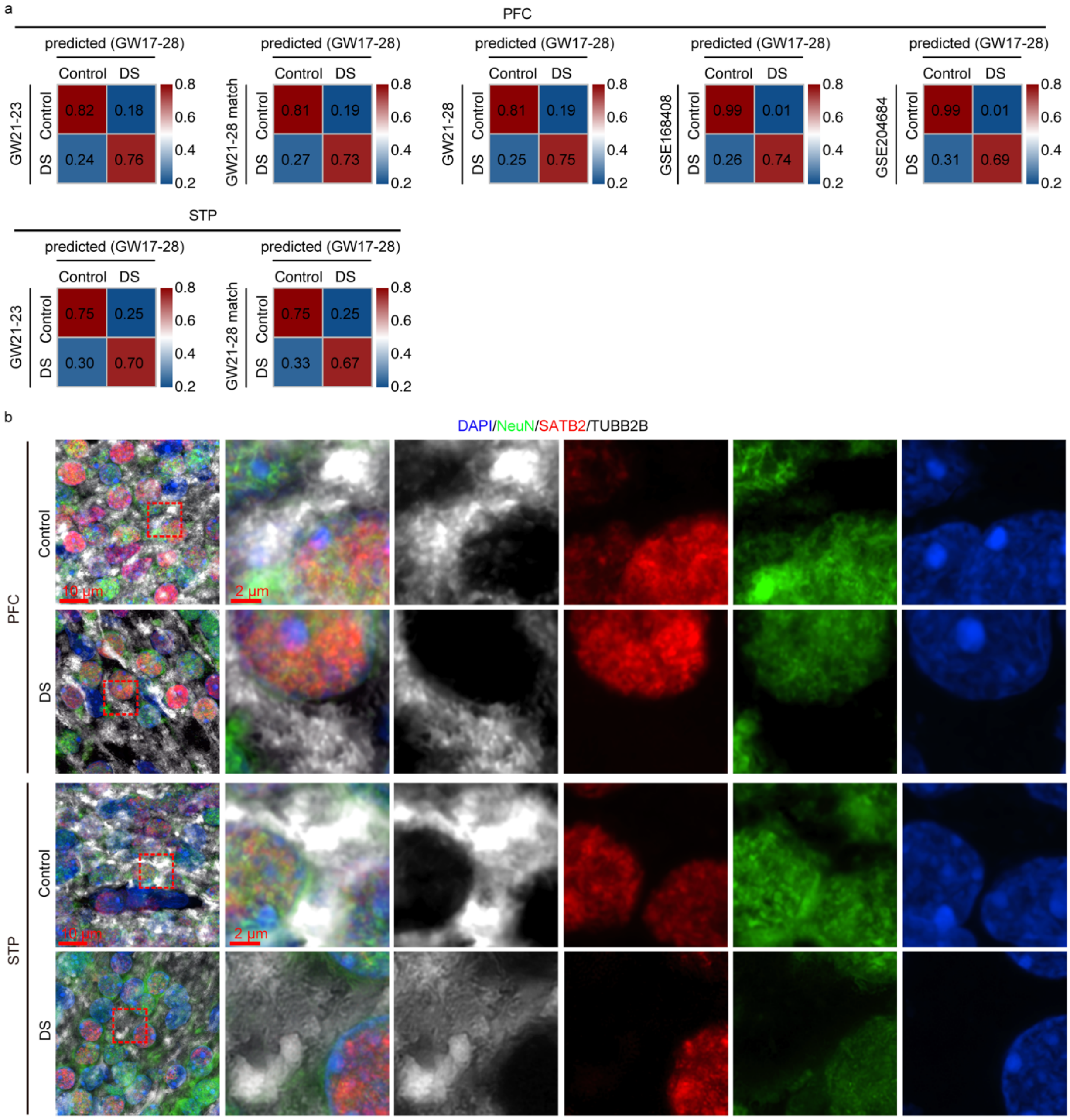

Supplementary Fig. 8. Stratified Disease Prediction Accuracy and High-Resolution Visualization of Neuronal Subtypes in PFC and STP at GW23. a Up, Confusion matrices of disease prediction performance

for different stratified data and two publicly published datasets of PFC. Below, Confusion matrices of disease prediction performance for different stratified data of STP. **b** Representative ultra-high resolution fields of *TUBB2B(White)/ NeuN(green) /SATB2 (red)* positive cells in the PFC and STP from GW23 of control and DS. Blue, DAPI. Scale bar: low magnification, 10 μm; high magnification, 2 μm.

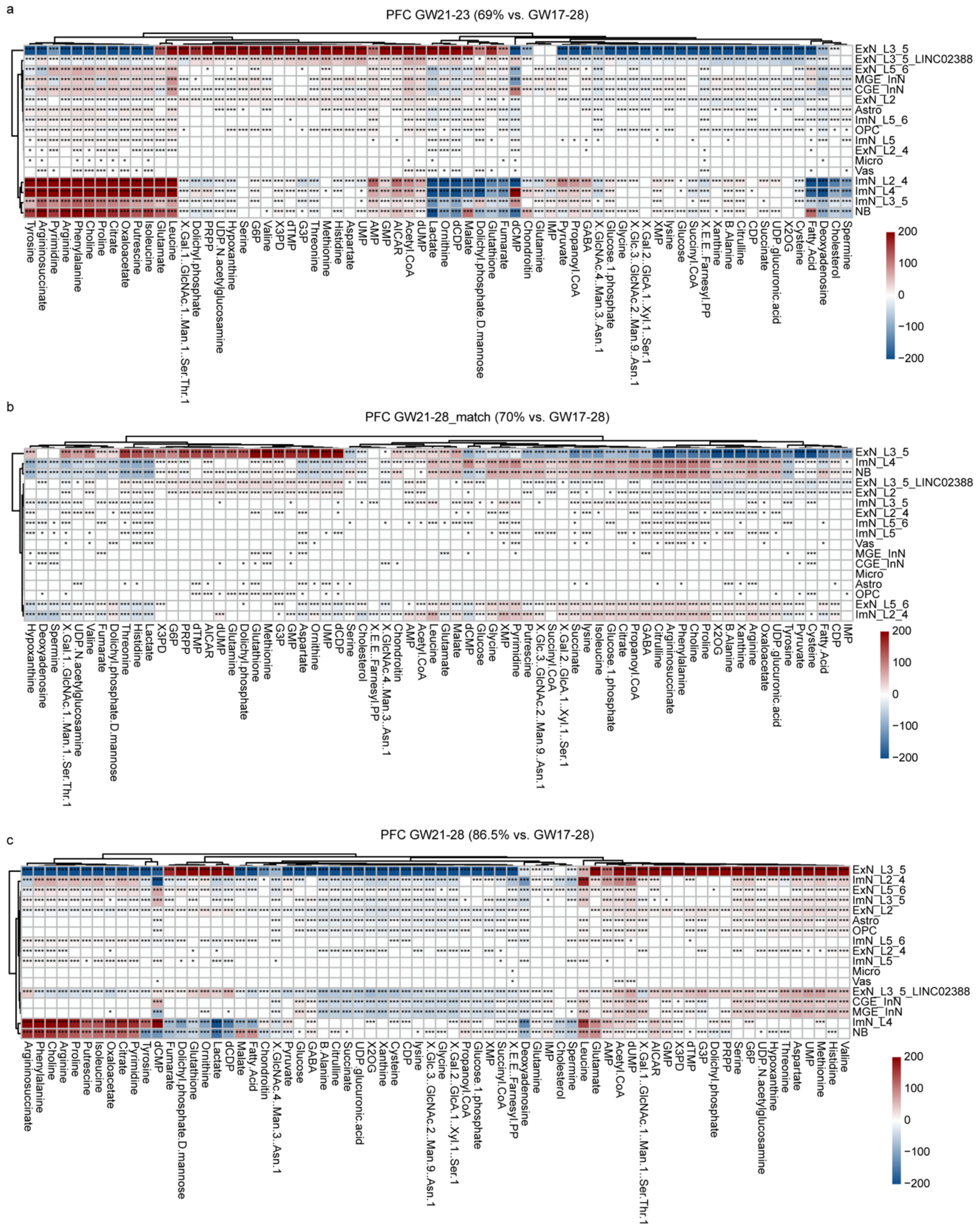

Supplementary Fig. 9. Metabolite analysis in the hierarchical data from PFC. a-c 68 metabolites showed differential expression in different cell types from GW 21-23 (**a**) , GW 21-28 match (**b**) and GW 21-28 (**c**) data (two-sided Wilcoxon rank-sum test, *$p < 0.05$, **$p < 0.01$, ***$p < 0.001$). Red indicates upregulation in DS, and

blue indicates downregulation. Percentages indicate the consistency rate of each stratified subgroup compared to the full dataset (GW17–28).

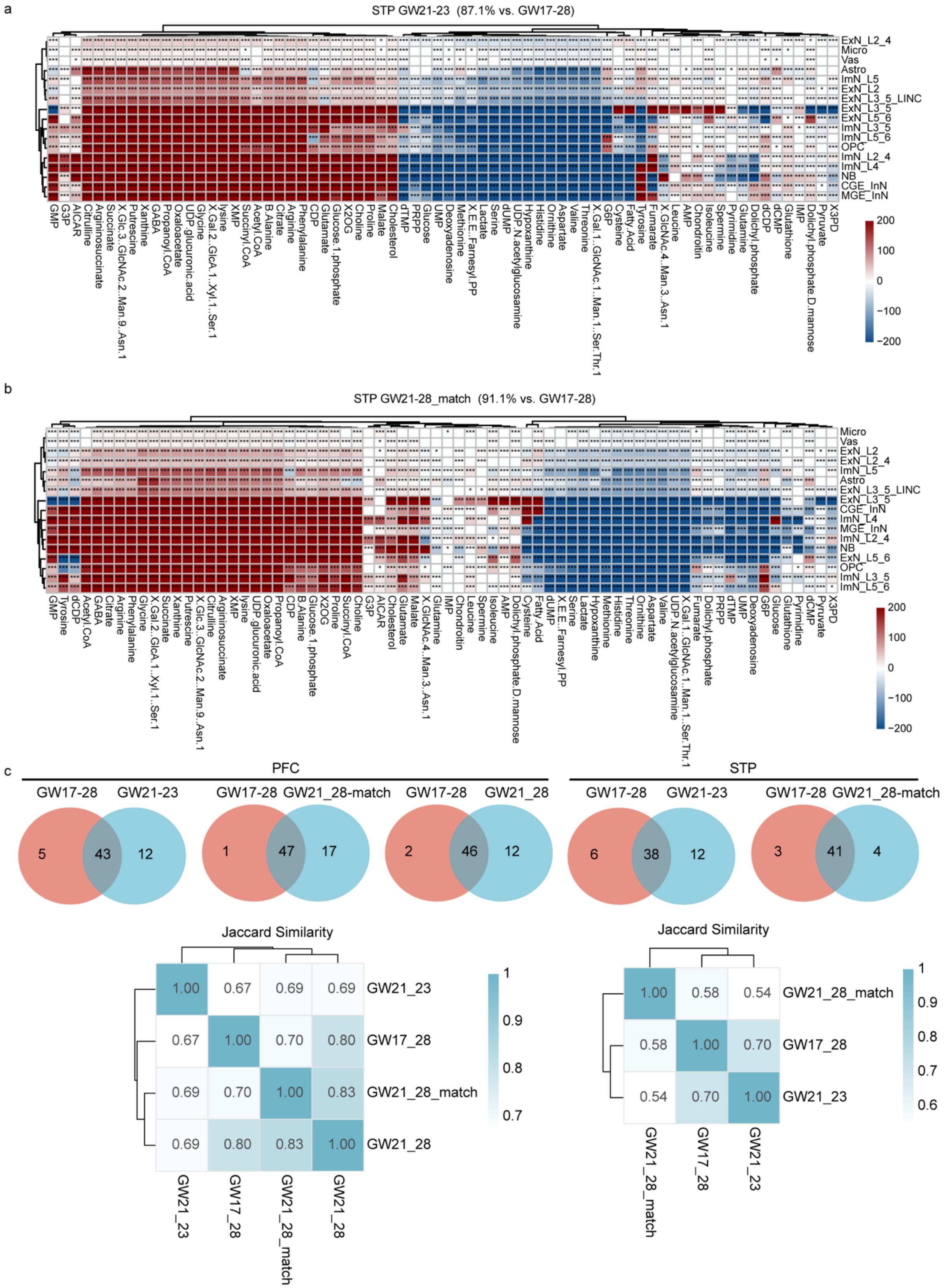
a
STP GW21-23 (87.1% vs. GW17-28)
ExN_L2_4
Micro
Vas
Astro
ImN_L5
ExN_L2
ExN_L3_5_LINC
ExN_L3_5
ExN_L5_6
ImN_L3_5
ImN_L5_6
OPC
ImN_L2_4
ImN_L4
NB
CGE_InN
MGE_InN
GMP
G3P
AICAR
Citrulline
Argininosuccinate
Succinate
X.Glc.3..GlcNAc.2..Man.9..Asn.1
Putrescine
Xanthine
GABA
Propanoyl.CoA
Oxaloacetate
UDP.glucuronic.acid
Glycine
X.Gal.2..GlcA.1..Xyl.1..Ser.1
lysine
XMP
Succinyl.CoA
Acetyl.CoA
B.Alanine
Citrate
Arginine
Phenylalanine
CDP
Glutamate
Glucose.1.phosphate
X2OG
Choline
Proline
Malate
Cholesterol
dTMP
PRPP
Glucose
UMP
Deoxyadenosine
Methionine
X.E.E..Farnesyl.PP
Lactate
Serine
dUMP
UDP.N.acetylglucosamine
Hypoxanthine
Histidine
Ornithine
Aspartate
Valine
Threonine
X.Gal.1..GlcNAc.1..Man.1..Ser.Thr.1
G6P
Cysteine
Fatty.Acid
Tyrosine
Fumarate
X.GlcNAc.4..Man.3..Asn.1
Leucine
AMP
Chondroitin
Isoleucine
Spermine
Pyrimidine
Glutamine
Dolichyl.phosphate
dCDP
dCMP
Glutathione
IMP
Dolichyl.phosphate.D.mannose
Pyruvate
X3PD
200
100
0
−100
−200
b
STP GW21-28_match (91.1% vs. GW17-28)
Micro
Vas
ExN_L2
ExN_L2_4
ImN_L5
Astro
ExN_L3_5_LINC
ExN_L3_5
CGE_InN
ImN_L4
MGE_InN
ImN_L2_4
NB
ExN_L5_6
OPC
ImN_L3_5
ImN_L5_6
GMP
Tyrosine
dCDP
Acetyl.CoA
GABA
Citrate
Arginine
Phenylalanine
Glycine
X.Gal.2..GlcA.1..Xyl.1..Ser.1
Xanthine
Putrescine
Succinate
X.Glc.3..GlcNAc.2..Man.9..Asn.1
Citrulline
Argininosuccinate
lysine
XMP
UDP.glucuronic.acid
Oxaloacetate
Propanoyl.CoA
CDP
B.Alanine
Glucose.1.phosphate
X2OG
Proline
Succinyl.CoA
Choline
G3P
AICAR
Cholesterol
Malate
Glutamate
X.GlcNAc.4..Man.3..Asn.1
Glutamine
IMP
Chondroitin
Leucine
Isoleucine
Spermine
AMP
Dolichyl.phosphate.D.mannose
Cysteine
Fatty.Acid
dUMP
X.E.E..Farnesyl.PP
Serine
Lactate
Hypoxanthine
Methionine
Histidine
Threonine
Ornithine
Aspartate
Valine
UDP.N.acetylglucosamine
X.Gal.1..GlcNAc.1..Man.1..Ser.Thr.1
Fumarate
Dolichyl.phosphate
PRPP
dTMP
UMP
Deoxyadenosine
G6P
Glucose
Glutathione
Pyrimidine
dCMP
Pyruvate
X3PD
200
100
0
−100
−200
c
PFC
STP
GW17-28
GW21-23
5
43
12
GW17-28
GW21_28-match
1
47
17
GW17-28
GW21_28
2
46
12
GW17-28
GW21-23
6
38
12
GW17-28
GW21_28-match
3
41
4
Jaccard Similarity
1.00 0.67 0.69 0.69 GW21_23
0.67 1.00 0.70 0.80 GW17_28
0.69 0.70 1.00 0.83 GW21_28_match
0.69 0.80 0.83 1.00 GW21_28
GW21_23
GW17_28
GW21_28_match
GW21_28
1
0.9
0.8
0.7
Jaccard Similarity
1.00 0.58 0.54 GW21_28_match
0.58 1.00 0.70 GW17_28
0.54 0.70 1.00 GW21_23
GW21_28_match
GW17_28
GW21_23
1
0.9
0.8
0.7
0.6

Supplementary Fig. 10. Metabolite analysis in the hierarchical data from STP. a-b 68 metabolites showed differential expression in different cell types from GW 21-23 (**a**) and GE 21-28 match data (**b**) (two-sided Wilcoxon rank-sum test, *$p < 0.05$, **$p < 0.01$, ***$p < 0.001$). Red indicates upregulation in DS, and blue indicates downregulation. **c** Venn diagram showing DEGs (FDR-adjusted p - value < 0.05, |log2FC| > 0.25) associated with lactylated proteins in the PFC and STP. Jaccard similarity analyses compared overlapped lactylation-regulated molecules between stratified subgroups to the full GW17–28 dataset. Percentages indicate the consistency rate of each stratified subgroup compared to the full dataset (GW17–28).

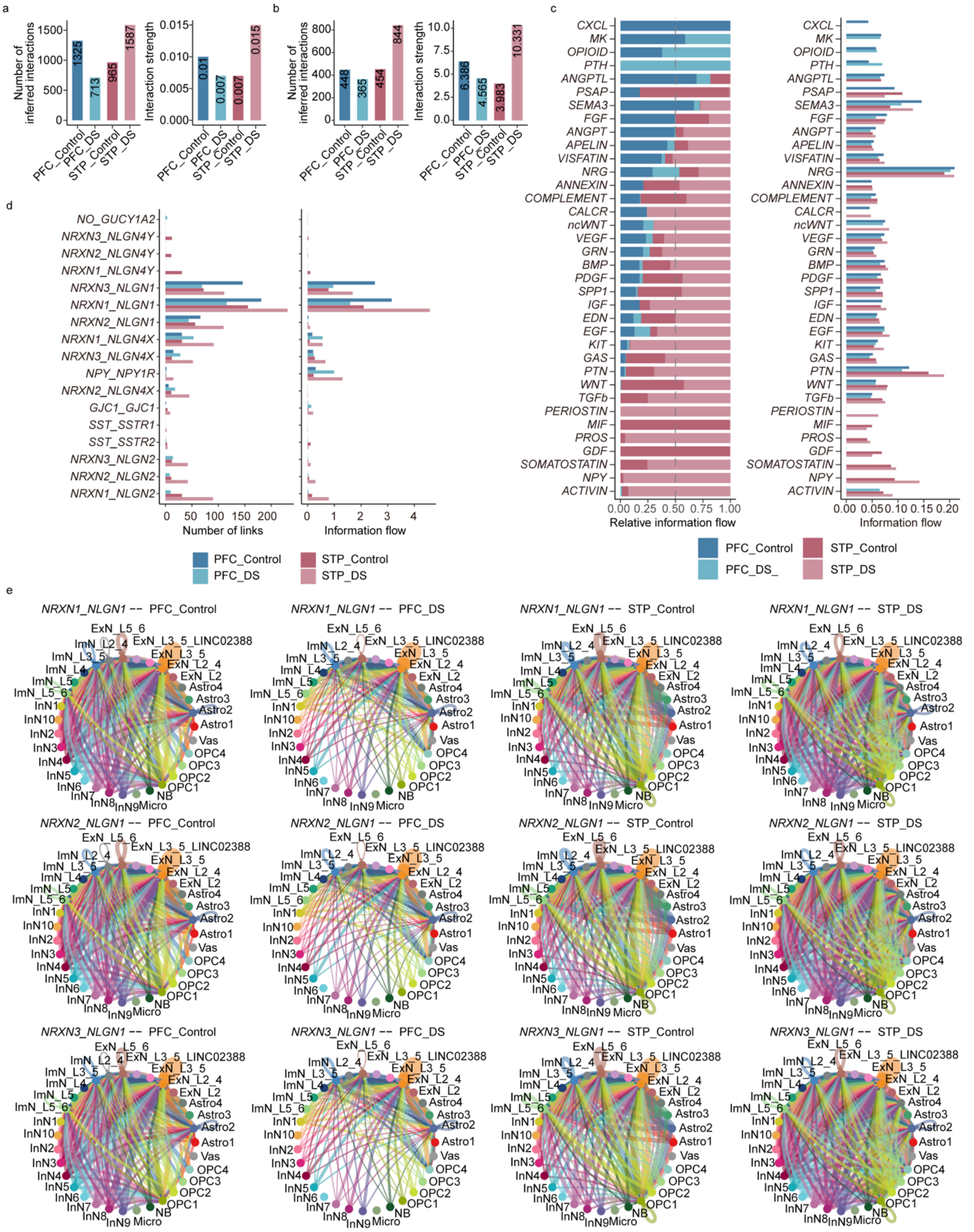

Supplementary Fig. 11. Comparison of cell communication and signaling pathway for control and DS from PFC and STP. a, b Cross-region comparison of inferred cell-cell interactions and interaction strengths by CellChat (a) and NeuroChat (b). Left: Bar plot showing the number of inferred cell-cell interactions for control

and DS from PFC and STP. Right: Bar plot showing the average interaction strength for each group. **c, d** Bar plots showing the strength and number of cell-cell interactions in control and DS from PFC and STP analysed by CellChat (c) and NeuroChat (d). **e** Comparison of cell–cell communication of *NRXN1-NLGN1*, *NRXN2-NLGN1 and NRXN3-NLGN1* signaling pathway in control and DS from PFC and STP.

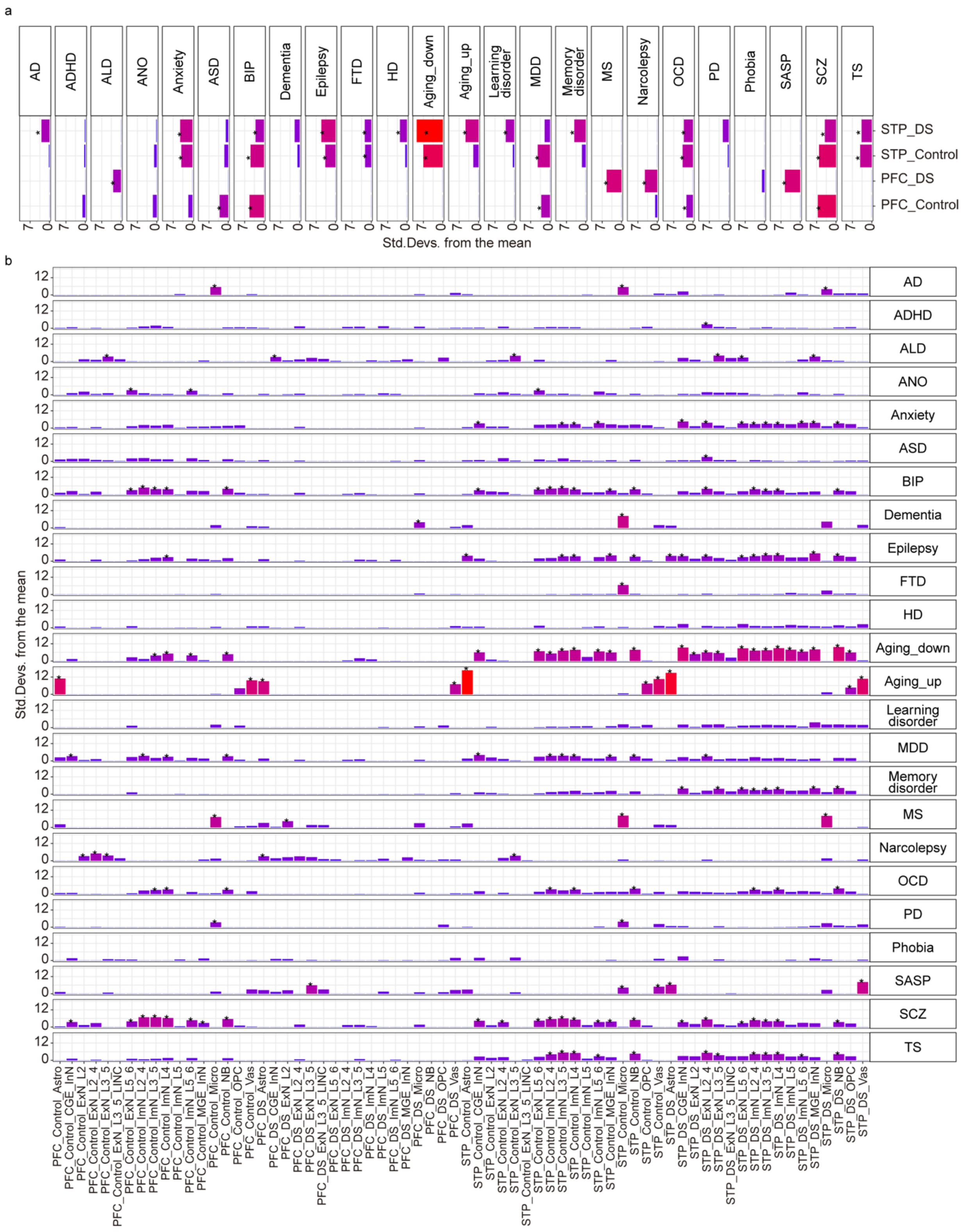

Supplementary Fig. 12. Enrichment analysis of neurological disease genes in control and DS from PFC and STP. a Group enrichment level of 24 gene sets associated with neurological diseases in the two cortex. Bar plot showing the standard derivation of a certain disease risk gene set, with region-related group indicated righg

the plot. Asterisks denote the BH-corrected p-value < 0.05 calculated using EWCE (Permutation Test). AD, Alzheimer's disease; ADHD, attention deficit hyperactivity disorder; ANO, anorexia nervosa; ASD, autism spectrum disorder; BIP, bipolar disorder; FTD, frontotemporal dementia; HD, Huntington's dementia; MDD, major depression disorder; MS, multiple sclerosis; OCD, obsessive–compulsive disorder; PD, Parkinson's disease; SASP, senescence-associated secretory phenotype; SCZ, schizophrenia; TOS, Tourette syndrome. **b** Cell types enrichment level of 24 gene sets associated with neurological diseases in the two cortex.

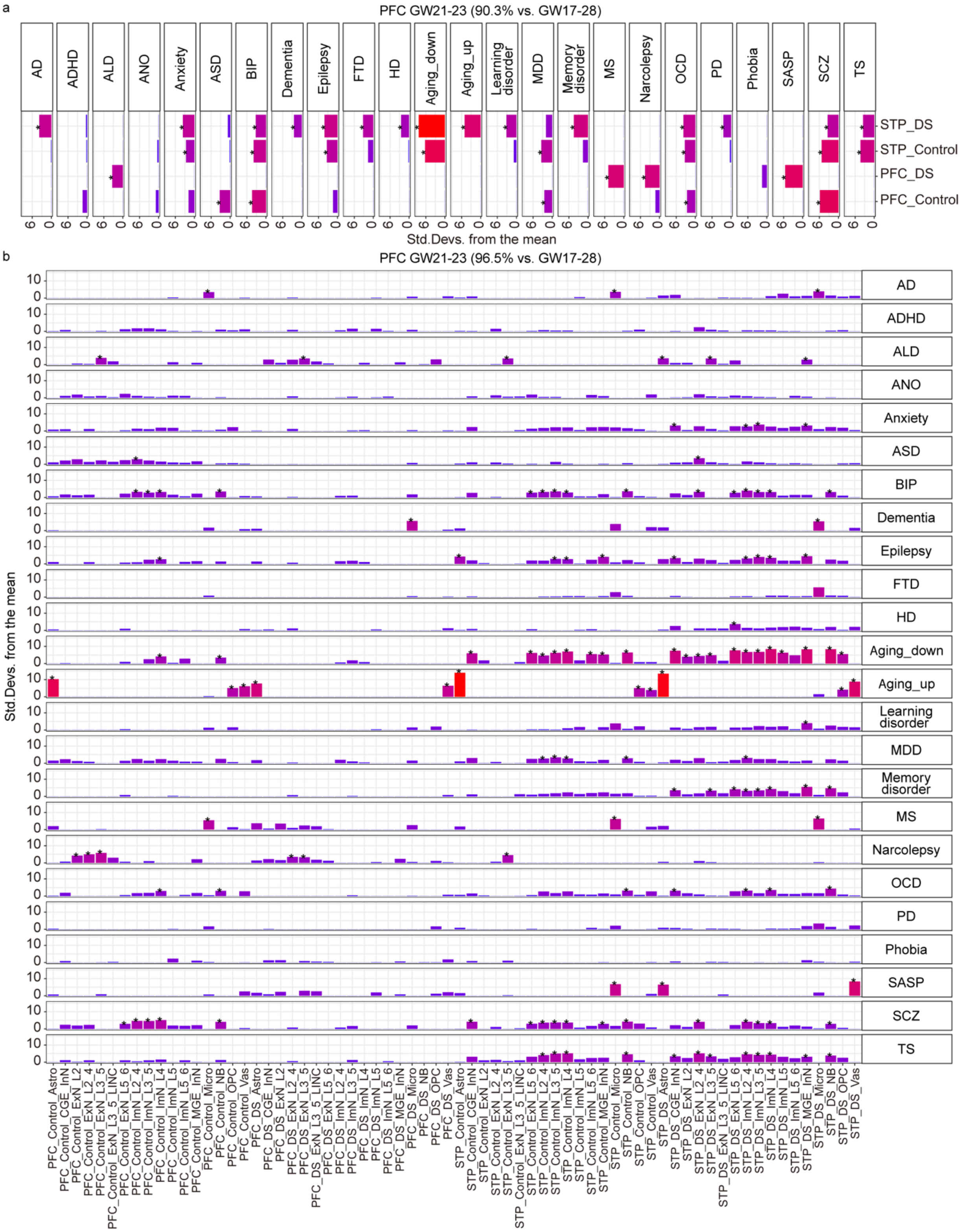

Supplementary Fig. 13. Enrichment analysis of neurological disease genes in control and DS from GW21-23 data. a Group enrichment level of 24 gene sets associated with neurological diseases in the two cortex. Bar plot showing the standard derivation of a certain disease risk gene set, with region-related group indicated righg

the plot. Asterisks denote the BH-corrected *p*-value < 0.05 calculated using EWCE (Permutation Test). **b** Cell types enrichment level of 24 gene sets associated with neurological diseases in the two cortex. Percentages indicate the consistency rate of each stratified subgroup compared to the full dataset (GW17–28).

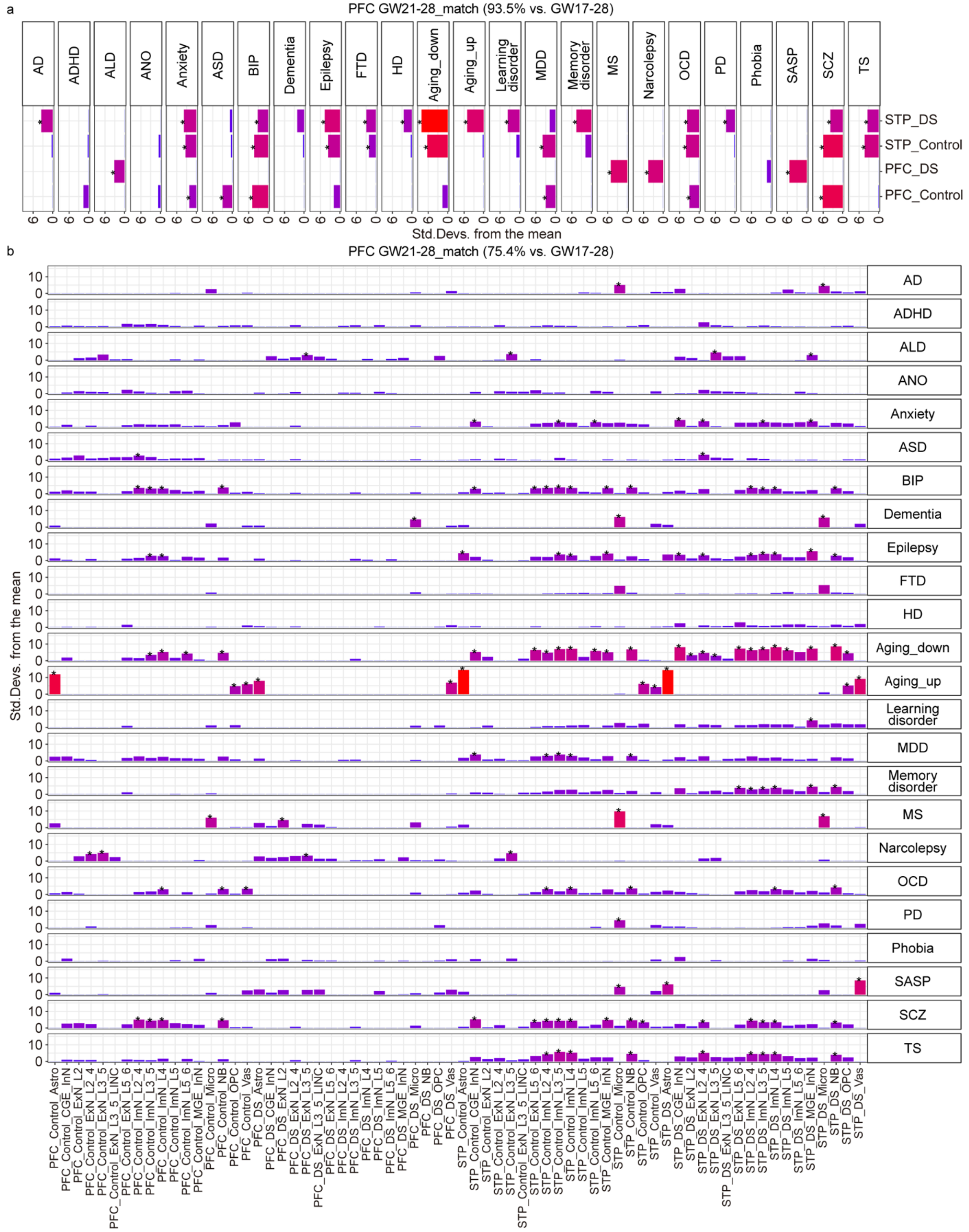

Supplementary Fig. 14. Enrichment analysis of neurological disease genes in control and DS from GW21-28 match data. a Group enrichment level of 24 gene sets associated with neurological diseases in the two cortex. Bar plot showing the standard derivation of a certain disease risk gene set, with region-related group indicated righg the plot. Asterisks denote the BH-corrected p-value < 0.05 calculated using EWCE (Permutation Test). **b** Cell types enrichment level of 24 gene sets associated with neurological diseases in the two cortex. Percentages indicate the consistency rate of each stratified subgroup compared to the full dataset (GW17–28).

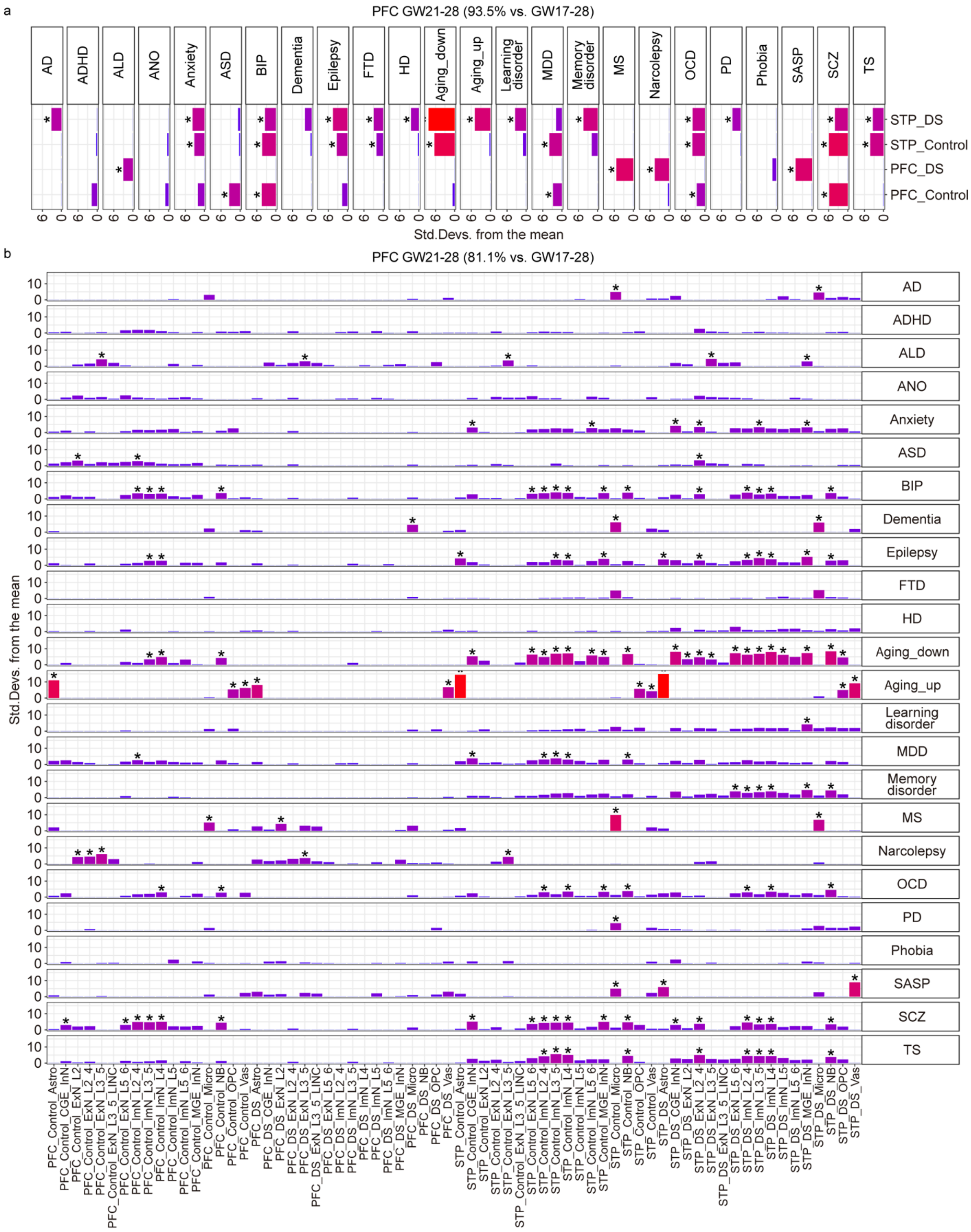

Supplementary Fig. 15. Enrichment analysis of neurological disease genes in control and DS from GW21-28 data. a Group enrichment level of 24 gene sets associated with neurological diseases in the two cortex. Bar plot showing the standard derivation of a certain disease risk gene set, with region-related group indicated righg

the plot. Asterisks denote the BH-corrected p-value < 0.05 calculated using EWCE (Permutation Test). **b** Cell types enrichment level of 24 gene sets associated with neurological diseases in the two cortex. Percentages indicate the consistency rate of each stratified subgroup compared to the full dataset (GW17–28).
