# Supplementary material for: Mid-gestational cell-type-specific transcriptomic signatures in the prefrontal and superior temporal cortex in Down syndrome
Source: Nat Commun. 2025 Dec 11;16:11249. doi: 10.1038/s41467-025-66109-9 (PMC12717210; doi:10.1038/s41467-025-66109-9)
Supplement: Supplementary file 2 — Description of Additional Supplementary Files [file 41467_2025_66109_MOESM2_ESM.pdf]

Description of Additional Supplementary Files

Supplementary Data 1: Demographic information and summary statistics for single nucleus libraries.

Supplementary Data 2: Marker genes for clusters and cell types.

Supplementary Data 3: Cell number and proportions for cell types and subclusters.

Supplementary Data 4: Marker genes of different cell types in neocortex of fetuses. Two-sided Wilcoxon rank-sum test with Benjamini-Hochberg correction for multiple testing. Only genes with logFC > 0.25 and expression in >25% of cells were retained.

Supplementary Data 5: DEGs for each cell type (adjust.p.value < 0.05, |logFC| > 0.25). Two-sided likelihood ratio test based on a generalized linear model (MAST), with covariate adjustment for library size, mitochondrial gene content, and sex. P-values were corrected for multiple comparisons using the Benjamini-Hochberg method.

Supplementary Data 6: Female only DEGs/global DEGs (%). Two-sided likelihood ratio test based on MAST. P-values were corrected for multiple comparisons using the Benjamini-Hochberg method.

Supplementary Data 7: The proportion of DEGs from different hierarchical data in the global DEGs (%). Two-sided likelihood ratio test based on MAST. P-values were corrected for multiple comparisons using the Benjamini-Hochberg method.

Supplementary Data 8: The differentially expressed chr21 gene in the hierarchical data. Two-sided likelihood ratio test based on MAST. P-values were corrected for multiple comparisons using the Benjamini-Hochberg method.

Supplementary Data 9: Genes perturbed by the virtual-KO of RUNX1 and APP (FDR < 0.05). P-values were computed using a Chi-square distribution with one degree of freedom, based on the fold change relative to expected values. P-values were adjusted for multiple testing using the Benjamini-Hochberg method.

Supplementary Data 10: DEGs of ExNs and InNs in old and young group (adjust.p.value < 0.05, |logFC| > 0.25). Two-sided likelihood ratio test based on MAST. P-values were corrected for multiple comparisons using the Benjamini-Hochberg method.

Supplementary Data 11: DEGs of blood from 304 individuals with DS versus 96 euploid controls (adjust.p.value < 0.05, |logFC| > 0.5). Wald test, p-values were adjusted using the Benjamini-Hochberg method to control the FDR.

Supplementary Data 12: Neuron migration related genes used in this study.

Supplementary Data 13: Genes perturbed by the virtual-KO of DSCAM, RUNX1, TIAM1, TUBA1A, TUBA1B, TUBB2A and TUBB2B in PFC ExNs (FDR < 0.05). P-values were computed using a Chi-square distribution with one degree of freedom, based on the fold change relative to expected values. P-values were adjusted for multiple testing using the Benjamini-Hochberg method.

Supplementary Data 14: Genes perturbed by the virtual-KO of APP, STMN1, STMN2, TUBA1A, TUBA1B, TUBB2A and TUBB2B in STP ExNs (FDR < 0.05). P-values were computed using a Chi-square

distribution with one degree of freedom, based on the fold change relative to expected values. P-values were adjusted for multiple testing using the Benjamini-Hochberg method.

Supplementary Data 15: Lactylation related proteins in cortical neurons. Supplementary Data 16 Genes perturbed by the virtual-KO of ERBB4 and NLGN1 in PFC and STP ExNs (FDR < 0.05). Pvalues were computed using a Chi-square distribution with one degree of freedom, based on the fold change relative to expected values. P-values were adjusted for multiple testing using the Benjamini-Hochberg method.

Supplementary Data 17: Disease risk gene sets used in this study. Supplementary Data 18 DS-related cell migration gene modules (DSMM). Supplementary Data 19 The antibody information used in this study.

Supplementary Data 18: DS-related cell migration gene modules (DSMM).

Supplementary Data 19: The antibody information used in this study
