# Supplementary material for: Mid-gestational cell-type-specific transcriptomic signatures in the prefrontal and superior temporal cortex in Down syndrome
Source: Nat Commun. 2025 Dec 11;16:11249. doi: 10.1038/s41467-025-66109-9 (PMC12717210; doi:10.1038/s41467-025-66109-9)
Supplement: Supplementary file 4 — Reporting Summary [file 41467_2025_66109_MOESM4_ESM.pdf]

nature portfolio

Corresponding author(s): LLXiong

Last updated by author(s): Sep 26, 2025

Reporting Summary

Nature Portfolio wishes to improve the reproducibility of the work that we publish. This form provides structure for consistency and transparency in reporting. For further information on Nature Portfolio policies, see our Editorial Policies and the Editorial Policy Checklist.

Statistics

For all statistical analyses, confirm that the following items are present in the figure legend, table legend, main text, or Methods section.

n/a	Confirmed	
☐	☒	The exact sample size (*n*) for each experimental group/condition, given as a discrete number and unit of measurement
☐	☒	A statement on whether measurements were taken from distinct samples or whether the same sample was measured repeatedly
☐	☒	The statistical test(s) used AND whether they are one- or two-sided<br>*Only common tests should be described solely by name; describe more complex techniques in the Methods section.*
☐	☒	A description of all covariates tested
☐	☒	A description of any assumptions or corrections, such as tests of normality and adjustment for multiple comparisons
☐	☒	A full description of the statistical parameters including central tendency (e.g. means) or other basic estimates (e.g. regression coefficient) AND variation (e.g. standard deviation) or associated estimates of uncertainty (e.g. confidence intervals)
☐	☒	For null hypothesis testing, the test statistic (e.g. *F*, *t*, *r*) with confidence intervals, effect sizes, degrees of freedom and *P* value noted<br>*Give P values as exact values whenever suitable.*
☒	☐	For Bayesian analysis, information on the choice of priors and Markov chain Monte Carlo settings
☐	☒	For hierarchical and complex designs, identification of the appropriate level for tests and full reporting of outcomes
☐	☒	Estimates of effect sizes (e.g. Cohen's *d*, Pearson's *r*), indicating how they were calculated

Our web collection on statistics for biologists contains articles on many of the points above.

Software and code

Policy information about availability of computer code

Data collection	Single-cell RNA-seq data were acquired using the Cell Ranger soft ware (version 7.1) and CeleScope (version 1.12.0).<br>IHC images were acquired using the Nikon AXR NSPARC image software.
Data analysis	General data analysis, statistical tests, and plotting: R 4.2.2 with the packages Seurat (4.2.0), DoubletFinder (v2.0.3), ggplot2 (3.4.3), dplyr (1.1.4), ComplexHeatmap (2.14.0), AnnotationDbi (1.60.0), org.Hs.eg.db (3.16.0), EWCE (1.6.0), cowplot (1.1.1), patchwork (1.1.2), MySeuratWrappers (0.1.0), scRNAtoolVis (0.0.7), readxl (1.4.1), EnrichR (3.2), aPEAR (1.0.0), randomForest (4.7-1.1), caret (6.0-93), pROC (1.18.0), modeldata (1.2.0), tidymodels (1.1.1), recipes (1.0.9), h2o (3.44.0.2), cacoa (0.4.0), conos (1.5.2), Matrix (1.5-4), ggrastr (1.0.2), Cairo (1.6-0), sccore (1.0.5), ggsignif (0.6.4), monocle (2.26.0), scTenifoldKnk (1.0.2), scTenifoldNet (1.3), ClusterGVis (0.1.1), WGCNA (1.71), MAST (1.24.0), nichenetr (2.2.0), CellChat (1.6.1), CytoTRACE2 (1.0.0). Python 3.8.17 with the packages samtools (1.6), numpy (1.24.4), scDRS (1.0.3), velocyto (0.17.17), pyscenic (0.12.1),<br>IHC images were analyzed in Fiji Image J software (version 2.3.0).

For manuscripts utilizing custom algorithms or software that are central to the research but not yet described in published literature, software must be made available to editors and reviewers. We strongly encourage code deposition in a community repository (e.g. GitHub). See the Nature Portfolio guidelines for submitting code & software for further information.

Data

Policy information about availability of data

All manuscripts must include a data availability statement. This statement should provide the following information, where applicable:

- Accession codes, unique identifiers, or web links for publicly available datasets
- A description of any restrictions on data availability
- For clinical datasets or third party data, please ensure that the statement adheres to our policy

All sequencing data generated have been deposited in Genome Sequence Archive (GSA): HRA

Research involving human participants, their data, or biological material

Policy information about studies with human participants or human data. See also policy information about sex, gender (identity/presentation), and sexual orientation and race, ethnicity and racism.

Reporting on sex and gender	We performed snRNA-seq on 22 postmortem brain tissue samples from 4 fetuses (male:female ratios 1:3) with DS and 11 controls (male:female ratios 7:4).
Reporting on race, ethnicity, or other socially relevant groupings	No reporting on race, ethnicity or other socially relevant groupings were used.
Population characteristics	We performed snRNA-seq on 22 postmortem brain tissue samples from 4 fetuses (male:female ratios 1:3) with DS and 11 controls (male:female ratios 7:4). Trisomy 21 was confirmed in all fetuses with DS through karyotype testing.
Recruitment	No participants were recruited.
Ethics oversight	This study has been clinically registered (Trial registration: ChiCTR, ChiCTR2300070041), and human fetal sample collection and research analysis was approved by the Affiliated Hospital of Zunyi Medical University (Approval No. KLL-2022-446). Informed consent for fetal tissue procurement and research was obtained from the patient after her decision to legally terminate her pregnancy but before the abortive procedure. Fetal brain tissue samples were collected after the donor signed an informed consent document that was in strict observance of the legal and institutional ethical regulations for elective pregnancy termination specimens at Affiliated Hospital of Zunyi Medical University. All samples used in these studies had not been involved in any other procedures. All the protocols were in compliance with the 'Interim Measures for the Administration of Human Genetic Resources' administered by the Chinese Ministry of Science and Technology.

Note that full information on the approval of the study protocol must also be provided in the manuscript.

Field-specific reporting

Please select the one below that is the best fit for your research. If you are not sure, read the appropriate sections before making your selection.

☒ Life sciences ☐ Behavioural & social sciences ☐ Ecological, evolutionary & environmental sciences

For a reference copy of the document with all sections, see nature.com/documents/nr-reporting-summary-flat.pdf

Life sciences study design

All studies must disclose on these points even when the disclosure is negative.

Sample size	Samples sizes for each experiment are clearly delineated in the manuscript.<br>The sample sizes for all experiments in this study were determined based on the number of clinically available and collectable samples. No formal sample size calculation was performed. Instead, the sample sizes were chosen to ensure sufficient representation and statistical power within the constraints of the available clinical samples.
Data exclusions	Low quality snRNA-seq libraries were excluded and the exclusion criteria are described in the manuscript as follows. We only retained cells with the number of detected genes greater than 200 and the percentage of detected mitochondrial genes less than 5%. Doublets were detected using DoubletFinder. After sample integration and clustering, clusters lacking specific marker genes, with relatively low gene content and high mitochondrial ratios were discarded.
Replication	Immunostaining was performed on additional 3 human tissue samples to confirm findings. All attempts at replication were successful.
Randomization	No randomization was used as there were no treated versus untreated groups; this work involved prospectively collecting tissue for single cell gene expression assays. However, control and DS sample were randomly assigned to the different sets of experiments (single cell RNA-seq and IHC).
Blinding	Blinding was not feasible, as knowledge of the experimental conditions was required during the data collection and analyses.

Reporting for specific materials, systems and methods

We require information from authors about some types of materials, experimental systems and methods used in many studies. Here, indicate whether each material, system or method listed is relevant to your study. If you are not sure if a list item applies to your research, read the appropriate section before selecting a response.

Materials & experimental systems

n/a	Involved in the study	
☐	☒	Antibodies
☒	☐	Eukaryotic cell lines
☒	☐	Palaeontology and archaeology
☒	☐	Animals and other organisms
☒	☐	Clinical data
☒	☐	Dual use research of concern
☒	☐	Plants

Methods

n/a	Involved in the study	
☒	☐	ChIP-seq
☒	☐	Flow cytometry
☒	☐	MRI-based neuroimaging

Antibodies

Antibodies used

REAGENT; SOURCE; CATALOG#; DILUTION; LINK
Rabbit anti-FOXP2; abcam; Cat# ab16046 ; 1:500; https://www.abcam.cn/products/primary-antibodies/foxp2-antibody-ab16046.html
Mouse anti-SATB2; abcam; Cat# ab51502; 1:300; https://www.abcam.cn/products/primary-antibodies/satb1--satb2-antibody-satba4b10-c-terminal-ab51502.html
Rabbit anti-TUBB2; Univ; Cat# ABS101264; 1:300; https://www.univ-bio.com/tbb2b/abs101264-50ul.html
Mouse anti-Neun; abcam; Cat# ab104224; 1:300; https://www.abcam.cn/products/primary-antibodies/neun-antibody-1b7-neuronal-marker-ab104224.html
Rabbit anti-BCAS1 ; Thermo Fisher; Cat# PA5-20904; 1:1000; https://www.thermofisher.cn/cn/zh/antibody/product/BCAS1-Antibody-Polyclonal/PA5-20904
Rabbit anti-Olig2; abcam; Cat# ab109186 ; 1:400; https://www.abcam.cn/products/primary-antibodies/olig2-antibody-epr2673-ab109186.html
Mouse anti-GFAP; Servicebio;Cat# GB12096; 1:2000; https://www.service-bio.com/goodsdetail?id=600
Mouse anti-ID2; abclonal; Cat# MA5-32891; 1:200; https://www.thermofisher.cn/cn/zh/antibody/product/ID2-Antibody-clone-A4-D4-Monoclonal/MA5-32891?imageId=518383

Validation

All the above are well characterized commercial antibodies. For each one, the specificity has been tested by the manufacturer and verified independently by previous published studies. Validation profiles and relevant citations can be found in the links provided.

Plants

Seed stocks

Report on the source of all seed stocks or other plant material used. If applicable, state the seed stock centre and catalogue number. If plant specimens were collected from the field, describe the collection location, date and sampling procedures.

Novel plant genotypes

Describe the methods by which all novel plant genotypes were produced. This includes those generated by transgenic approaches, gene editing, chemical/radiation-based mutagenesis and hybridization. For transgenic lines, describe the transformation method, the number of independent lines analyzed and the generation upon which experiments were performed. For gene-edited lines, describe the editor used, the endogenous sequence targeted for editing, the targeting guide RNA sequence (if applicable) and how the editor was applied.

Authentication

Describe any authentication procedures for each seed stock used or novel genotype generated. Describe any experiments used to assess the effect of a mutation and, where applicable, how potential secondary effects (e.g. second site T-DNA insertions, mosiacism, off-target gene editing) were examined.
